# Propane-2,2-diyl di-*p*-phenyl­ene dibenzoate

**DOI:** 10.1107/S1600536807065531

**Published:** 2007-12-12

**Authors:** Tomislav Portada, Nenad Judaš

**Affiliations:** a Department of Organic Chemistry and Biochemistry, Ruđer Bošković Institute, PO Box 180, HR-10002 Zagreb, Croatia; b Department of Chemistry, Laboratory of General and Inorganic Chemistry, Faculty of Science, University of Zagreb, Horvatovac 102a, HR-10000 Zagreb, Croatia

## Abstract

The V-shaped propeller-like mol­ecule of the title compound, C_29_H_24_O_4_, does not exhibit crystallographic twofold symmetry as the two benzene rings are twisted asymmetrically with respect to both the central propyl plane and the benzo­yloxy groups [4.6 (2), 43.6 (2)° and 45.07 (8), 69.50 (8)°]. In the crystal structure, centrosymmetrically related mol­ecules form a dimer through C—H⋯π inter­molecular inter­actions.

## Related literature

For related literature, see: Perez & Scaringe (1987 ▶); Toda *et al.* (1988 ▶); Bocelli & Cantoni (1989 ▶); Casarini *et al.* (1995 ▶); Williams (1966 ▶). For bond-length data, see: Allen *et al.* (1987 ▶).
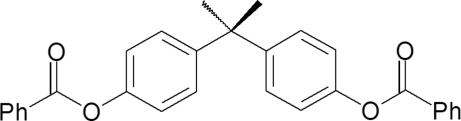


## Experimental

### 

#### Crystal data

C_29_H_24_O_4_
*M* *_r_* = 436.48
Monoclinic, 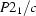

*a* = 8.7298 (2) Å
*b* = 21.4202 (4) Å
*c* = 12.6693 (3) Å
β = 104.291 (2)°
*V* = 2295.77 (9) Å^3^
*Z* = 4
Mo *K*α radiation
μ = 0.08 mm^−1^
*T* = 293 (2) K
0.68 × 0.46 × 0.23 mm

#### Data collection

Oxford Diffraction Xcalibur CCD diffractometer
Absorption correction: none
23733 measured reflections
4033 independent reflections
2427 reflections with *I* > 2σ(*I*)
*R*_int_ = 0.023

#### Refinement

*R*[*F* ^2^ > 2σ(*F* ^2^)] = 0.039
*wR*(*F* ^2^) = 0.109
*S* = 1.03
4033 reflections
300 parameters
H-atom parameters constrained
Δρ_max_ = 0.14 e Å^−3^
Δρ_min_ = −0.15 e Å^−3^

### 

Data collection: *CrysAlis CCD* (Oxford Diffraction, 2003 ▶); cell refinement: *CrysAlis RED* (Oxford Diffraction, 2003 ▶); data reduction: *CrysAlis RED*; program(s) used to solve structure: *SHELXS97* (Sheldrick, 1997 ▶); program(s) used to refine structure: *SHELXL97* (Sheldrick, 1997 ▶); molecular graphics: *ORTEP-3 for Windows* (Farrugia, 1997 ▶) and *POV-RAY* (Persistence of Vision, 2004 ▶); software used to prepare material for publication: *WinGX* (Farrugia, 1999 ▶).

## Supplementary Material

Crystal structure: contains datablocks I, global. DOI: 10.1107/S1600536807065531/ci2535sup1.cif

Structure factors: contains datablocks I. DOI: 10.1107/S1600536807065531/ci2535Isup2.hkl

Additional supplementary materials:  crystallographic information; 3D view; checkCIF report

## Figures and Tables

**Table 1:** Hydrogen-bond geometry (Å, °)

*D*—H⋯*A*	*D*—H	H⋯*A*	*D*⋯*A*	*D*—H⋯*A*
C26—H26⋯*Cg*2^i^	0.93	2.86	3.743 (2)	160

Symmetry code: (i) 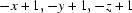. *Cg*2 is the centroid of the C10–C15 ring.

**Fig. 1.**
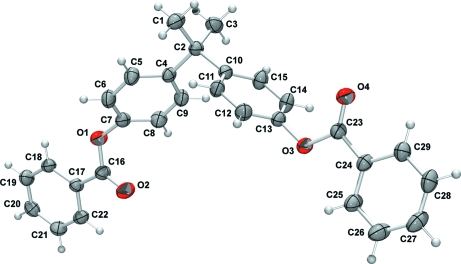
The molecular structure of the title compound, showing the atomic numbering scheme. Displacement ellipsoids are drawn at the 30% probability level and H atoms are shown as small spheres of arbitrary radius.

## supplementary crystallographic information

### Comment

The title compound was synthesized as a part of our work on organizing a workshop on parallel synthesis and combinatorial chemistry. The title compound belongs to the class of compounds that can be used for isomeric separation by crystalline inclusion or for studies on isomeric selectivity, host design and molecular recognition.

The molecular structure of the title compound is shown in Fig. 1. The V-shaped propeller-like molecule consists of several parts: central propyl part (C1—C3), two benzene rings (C4—C9 and C10—C15) and two benzoyloxy groups (O1/O2/C16—C22 and O3/O4/C23—C29). All bond lengths and angles fall within normal ranges (Allen *et al.*, 1987).

The molecule does not exhibit twofold symmetry as there is a characteristic asymmetric twist of the two benzene rings with respect to the C1/C2/C3 plane, which is best described by the torsion angles C5—C4—C2—C1 and C1—C2—C10—C11 of -4.6 (2)° and 43.6 (2)°, respectively. Further, the benzoyloxy moieties are also twisted asymmetrically with respect to the attached benzene rings. The dihedral angle between the C4—C9 and C17—C22 planes is 45.07 (8)° and that between the C10—C15 and C24—C29 planes is 69.50 (8)°.

In the crystal, the centrosymmetrically related molecules form a dimeric pair through C—H···π intermolecular interactions involving the C26—H26 group and the C10—C15 benzene ring (centroid *Cg*1) (Table 1).

### Experimental

Bisphenol A (500 mg, 2.19 mmol) was dissolved in an aqueous solution (3.0 ml) of 2 *M* sodium hydroxide (6 mmol) and benzoyl chloride (508 µ*L*, 616 mg, 4.38 mmol) was added to it. The reaction mixture was vigorously shaken for 20 min. The resulting white solid was filtered and rinsed with cold methanol and dried. Single crystals of the title compound were obtained by slow evaporation of a toluene solution.

### Refinement

H atoms were placed in calculated positions and included in the refinement using the riding-model approximation, with C—H distances of 0.93 Å for phenyl and 0.96 Å for methyl groups, and with *U*_iso_(H) = 1.2*U*_eq_(C) or 1.2*U*_eq_(C_methyl_). A rotating group model was used for the methyl groups.

### Crystal data

**Table:**

C_29_H_24_O_4_	*F*_000_ = 920
*M**_r_* = 436.48	*D*_x_ = 1.263 Mg m^−^^3^
Monoclinic, *P*2_1_/*c*	Melting point: 435 K
Hall symbol: -P 2ybc	Mo *K*α radiation λ = 0.71073 Å
*a* = 8.7298 (2) Å	Cell parameters from 238 reflections
*b* = 21.4202 (4) Å	θ = 8.4–23.2º
*c* = 12.6693 (3) Å	µ = 0.08 mm^−^^1^
β = 104.291 (2)º	*T* = 293 (2) K
*V* = 2295.77 (9) Å^3^	Prism, colourless
*Z* = 4	0.68 × 0.46 × 0.23 mm

### Data collection

**Table:**

Oxford Diffraction Xcalibur CCD diffractometer	2427 reflections with *I* > 2σ(*I*)
Radiation source: fine-focus sealed tube	*R*_int_ = 0.023
Monochromator: graphite	θ_max_ = 25.0º
*T* = 293(2) K	θ_min_ = 3.7º
ω scans	*h* = −10→10
Absorption correction: none	*k* = −25→25
23733 measured reflections	*l* = −15→15
4033 independent reflections	

### Refinement

**Table:**

Refinement on *F*^2^	Secondary atom site location: difference Fourier map
Least-squares matrix: full	Hydrogen site location: inferred from neighbouring sites
*R*[*F*^2^ > 2σ(*F*^2^)] = 0.039	H-atom parameters constrained
*wR*(*F*^2^) = 0.109	*w* = 1/[σ^2^(*F*_o_^2^) + (0.0623*P*)^2^] where *P* = (*F*_o_^2^ + 2*F*_c_^2^)/3
*S* = 1.03	(Δ/σ)_max_ = 0.001
4033 reflections	Δρ_max_ = 0.14 e Å^−^^3^
300 parameters	Δρ_min_ = −0.15 e Å^−^^3^
Primary atom site location: structure-invariant direct methods	Extinction correction: none

### Special details

**Table:**

Geometry. All e.s.d.'s (except the e.s.d. in the dihedral angle between two l.s. planes) are estimated using the full covariance matrix. The cell e.s.d.'s are taken into account individually in the estimation of e.s.d.'s in distances, angles and torsion angles; correlations between e.s.d.'s in cell parameters are only used when they are defined by crystal symmetry. An approximate (isotropic) treatment of cell e.s.d.'s is used for estimating e.s.d.'s involving l.s. planes.
Refinement. Refinement of *F*^2^ against ALL reflections. The weighted *R*-factor *wR* and goodness of fit *S* are based on *F*^2^, conventional *R*-factors *R* are based on *F*, with *F* set to zero for negative *F*^2^. The threshold expression of *F*^2^ > σ(*F*^2^) is used only for calculating *R*-factors(gt) *etc*. and is not relevant to the choice of reflections for refinement. *R*-factors based on *F*^2^ are statistically about twice as large as those based on *F*, and *R*- factors based on ALL data will be even larger.

### Fractional atomic coordinates and isotropic or equivalent isotropic displacement parameters (Å^2^)

**Table:**

	*x*	*y*	*z*	*U*_iso_*/*U*_eq_	
O1	−0.25033 (15)	0.50560 (5)	0.89440 (10)	0.0753 (4)	
O2	−0.13081 (17)	0.42428 (6)	0.83756 (12)	0.0937 (5)	
O3	0.41160 (13)	0.59632 (5)	0.44375 (9)	0.0644 (3)	
O4	0.41701 (15)	0.68081 (6)	0.34023 (10)	0.0768 (4)	
C1	0.0091 (2)	0.75543 (7)	0.71258 (14)	0.0723 (5)	
H1A	0.1022	0.7438	0.7670	0.108*	
H1B	−0.0694	0.7708	0.7474	0.108*	
H1C	0.0352	0.7875	0.6670	0.108*	
C2	−0.05579 (18)	0.69840 (7)	0.64295 (12)	0.0530 (4)	
C3	−0.2072 (2)	0.71965 (8)	0.56017 (15)	0.0752 (5)	
H3A	−0.1815	0.7516	0.5142	0.113*	
H3B	−0.2809	0.7359	0.5983	0.113*	
H3C	−0.2538	0.6847	0.5163	0.113*	
C4	−0.09977 (17)	0.64530 (7)	0.71197 (12)	0.0503 (4)	
C5	−0.0719 (2)	0.64800 (8)	0.82398 (14)	0.0681 (5)	
H5	−0.0208	0.6827	0.8605	0.082*	
C6	−0.1179 (2)	0.60034 (8)	0.88348 (15)	0.0730 (5)	
H6	−0.0981	0.6033	0.9589	0.088*	
C7	−0.1922 (2)	0.54922 (7)	0.83096 (15)	0.0594 (4)	
C8	−0.2206 (2)	0.54453 (8)	0.72020 (15)	0.0696 (5)	
H8	−0.2717	0.5096	0.6842	0.084*	
C9	−0.1726 (2)	0.59194 (8)	0.66261 (14)	0.0648 (5)	
H9	−0.1900	0.5879	0.5875	0.078*	
C10	0.06761 (19)	0.67327 (6)	0.58543 (12)	0.0492 (4)	
C11	0.2245 (2)	0.66805 (7)	0.64163 (13)	0.0611 (5)	
H11	0.2542	0.6811	0.7138	0.073*	
C12	0.3385 (2)	0.64411 (7)	0.59432 (14)	0.0625 (5)	
H12	0.4432	0.6414	0.6340	0.075*	
C13	0.2961 (2)	0.62455 (7)	0.48917 (13)	0.0528 (4)	
C14	0.1429 (2)	0.62839 (8)	0.43036 (14)	0.0664 (5)	
H14	0.1144	0.6146	0.3585	0.080*	
C15	0.0302 (2)	0.65297 (8)	0.47874 (14)	0.0652 (5)	
H15	−0.0739	0.6559	0.4380	0.078*	
C16	−0.2158 (2)	0.44437 (8)	0.89067 (14)	0.0623 (5)	
C17	−0.29534 (18)	0.40654 (7)	0.95880 (12)	0.0529 (4)	
C18	−0.37050 (19)	0.43330 (8)	1.03155 (13)	0.0603 (4)	
H18	−0.3700	0.4764	1.0401	0.072*	
C19	−0.4462 (2)	0.39608 (9)	1.09129 (15)	0.0706 (5)	
H19	−0.4965	0.4141	1.1405	0.085*	
C20	−0.4477 (2)	0.33262 (9)	1.07866 (15)	0.0704 (5)	
H20	−0.5011	0.3077	1.1180	0.084*	
C21	−0.3711 (2)	0.30582 (8)	1.00863 (15)	0.0745 (5)	
H21	−0.3699	0.2626	1.0017	0.089*	
C22	−0.2957 (2)	0.34237 (8)	0.94838 (15)	0.0692 (5)	
H22	−0.2445	0.3239	0.9002	0.083*	
C23	0.46431 (19)	0.62946 (8)	0.36846 (13)	0.0545 (4)	
C24	0.58527 (18)	0.59521 (7)	0.32854 (13)	0.0533 (4)	
C25	0.6445 (2)	0.53766 (8)	0.36993 (15)	0.0678 (5)	
H25	0.6056	0.5185	0.4239	0.081*	
C26	0.7605 (2)	0.50891 (8)	0.33132 (19)	0.0839 (6)	
H26	0.8003	0.4705	0.3596	0.101*	
C27	0.8177 (2)	0.53652 (9)	0.25142 (18)	0.0800 (6)	
H27	0.8959	0.5167	0.2256	0.096*	
C28	0.7604 (2)	0.59323 (10)	0.20925 (15)	0.0748 (5)	
H28	0.7989	0.6116	0.1545	0.090*	
C29	0.6450 (2)	0.62308 (8)	0.24852 (14)	0.0643 (5)	
H29	0.6075	0.6619	0.2211	0.077*	

### Atomic displacement parameters (Å^2^)

**Table:**

	*U*^11^	*U*^22^	*U*^33^	*U*^12^	*U*^13^	*U*^23^
O1	0.0976 (10)	0.0520 (7)	0.0952 (9)	0.0053 (6)	0.0600 (8)	0.0114 (6)
O2	0.1152 (11)	0.0755 (8)	0.1173 (11)	0.0196 (7)	0.0800 (10)	0.0121 (8)
O3	0.0735 (8)	0.0576 (7)	0.0742 (8)	0.0104 (5)	0.0414 (6)	0.0063 (6)
O4	0.0900 (9)	0.0587 (8)	0.0953 (9)	0.0110 (6)	0.0489 (8)	0.0088 (7)
C1	0.0843 (14)	0.0539 (10)	0.0911 (13)	−0.0044 (9)	0.0453 (11)	−0.0076 (9)
C2	0.0567 (10)	0.0476 (9)	0.0599 (10)	0.0015 (7)	0.0243 (8)	0.0020 (8)
C3	0.0714 (13)	0.0776 (12)	0.0822 (13)	0.0196 (9)	0.0294 (10)	0.0175 (10)
C4	0.0482 (9)	0.0530 (9)	0.0547 (10)	−0.0010 (7)	0.0219 (8)	−0.0030 (7)
C5	0.0836 (13)	0.0649 (11)	0.0604 (11)	−0.0192 (9)	0.0263 (10)	−0.0095 (9)
C6	0.0971 (15)	0.0724 (12)	0.0570 (11)	−0.0095 (10)	0.0332 (10)	−0.0013 (10)
C7	0.0646 (11)	0.0518 (10)	0.0726 (12)	0.0009 (8)	0.0375 (9)	0.0051 (9)
C8	0.0824 (13)	0.0571 (10)	0.0730 (13)	−0.0190 (9)	0.0265 (10)	−0.0072 (9)
C9	0.0796 (13)	0.0633 (11)	0.0544 (10)	−0.0125 (9)	0.0219 (9)	−0.0053 (9)
C10	0.0541 (10)	0.0454 (9)	0.0523 (9)	0.0001 (7)	0.0211 (8)	0.0036 (7)
C11	0.0635 (12)	0.0717 (11)	0.0493 (9)	0.0095 (9)	0.0163 (9)	−0.0046 (8)
C12	0.0528 (10)	0.0734 (11)	0.0609 (11)	0.0091 (8)	0.0135 (9)	0.0005 (9)
C13	0.0570 (11)	0.0495 (9)	0.0586 (11)	0.0047 (8)	0.0268 (9)	0.0025 (8)
C14	0.0669 (13)	0.0831 (12)	0.0537 (10)	−0.0057 (9)	0.0239 (10)	−0.0145 (9)
C15	0.0529 (11)	0.0858 (12)	0.0585 (11)	−0.0013 (9)	0.0166 (9)	−0.0089 (9)
C16	0.0648 (12)	0.0567 (11)	0.0724 (12)	0.0062 (9)	0.0305 (10)	0.0038 (9)
C17	0.0521 (10)	0.0501 (9)	0.0593 (10)	0.0030 (7)	0.0191 (8)	0.0036 (8)
C18	0.0692 (11)	0.0515 (9)	0.0652 (11)	0.0057 (8)	0.0263 (9)	0.0034 (8)
C19	0.0756 (13)	0.0744 (13)	0.0702 (12)	0.0073 (10)	0.0339 (10)	0.0103 (10)
C20	0.0711 (13)	0.0700 (13)	0.0709 (12)	−0.0083 (9)	0.0192 (10)	0.0171 (10)
C21	0.0941 (14)	0.0502 (10)	0.0807 (13)	−0.0034 (9)	0.0246 (12)	0.0034 (9)
C22	0.0837 (13)	0.0558 (11)	0.0751 (12)	0.0070 (9)	0.0328 (10)	−0.0006 (9)
C23	0.0582 (10)	0.0507 (10)	0.0582 (10)	−0.0061 (8)	0.0208 (8)	−0.0047 (8)
C24	0.0506 (10)	0.0542 (10)	0.0594 (10)	−0.0057 (7)	0.0219 (8)	−0.0098 (8)
C25	0.0724 (12)	0.0564 (11)	0.0862 (13)	−0.0029 (9)	0.0415 (10)	−0.0033 (9)
C26	0.0813 (14)	0.0607 (11)	0.1244 (17)	0.0049 (10)	0.0533 (13)	−0.0052 (11)
C27	0.0685 (13)	0.0770 (13)	0.1078 (16)	−0.0086 (10)	0.0469 (12)	−0.0285 (12)
C28	0.0671 (13)	0.0954 (15)	0.0715 (12)	−0.0142 (11)	0.0355 (10)	−0.0119 (11)
C29	0.0612 (11)	0.0725 (11)	0.0636 (11)	−0.0043 (9)	0.0239 (9)	−0.0004 (9)

### Geometric parameters (Å, °)

**Table:**

O1—C16	1.3492 (19)	C12—C13	1.358 (2)
O1—C7	1.4064 (18)	C12—H12	0.93
O2—C16	1.1981 (18)	C13—C14	1.363 (2)
O3—C23	1.3574 (18)	C14—C15	1.385 (2)
O3—C13	1.4144 (17)	C14—H14	0.93
O4—C23	1.1980 (18)	C15—H15	0.93
C1—C2	1.531 (2)	C16—C17	1.476 (2)
C1—H1A	0.96	C17—C18	1.381 (2)
C1—H1B	0.96	C17—C22	1.381 (2)
C1—H1C	0.96	C18—C19	1.375 (2)
C2—C10	1.539 (2)	C18—H18	0.93
C2—C4	1.540 (2)	C19—C20	1.368 (2)
C2—C3	1.540 (2)	C19—H19	0.93
C3—H3A	0.96	C20—C21	1.363 (2)
C3—H3B	0.96	C20—H20	0.93
C3—H3C	0.96	C21—C22	1.370 (2)
C4—C9	1.381 (2)	C21—H21	0.93
C4—C5	1.380 (2)	C22—H22	0.93
C5—C6	1.386 (2)	C23—C24	1.474 (2)
C5—H5	0.93	C24—C29	1.384 (2)
C6—C7	1.360 (2)	C24—C25	1.389 (2)
C6—H6	0.93	C25—C26	1.374 (2)
C7—C8	1.367 (2)	C25—H25	0.93
C8—C9	1.375 (2)	C26—C27	1.368 (3)
C8—H8	0.93	C26—H26	0.93
C9—H9	0.93	C27—C28	1.371 (2)
C10—C15	1.380 (2)	C27—H27	0.93
C10—C11	1.383 (2)	C28—C29	1.385 (2)
C11—C12	1.381 (2)	C28—H28	0.93
C11—H11	0.93	C29—H29	0.93
			
C16—O1—C7	120.72 (12)	C14—C13—O3	120.34 (15)
C23—O3—C13	117.50 (12)	C13—C14—C15	119.28 (16)
C2—C1—H1A	109.5	C13—C14—H14	120.4
C2—C1—H1B	109.5	C15—C14—H14	120.4
H1A—C1—H1B	109.5	C10—C15—C14	122.06 (17)
C2—C1—H1C	109.5	C10—C15—H15	119.0
H1A—C1—H1C	109.5	C14—C15—H15	119.0
H1B—C1—H1C	109.5	O2—C16—O1	122.91 (15)
C1—C2—C10	110.44 (13)	O2—C16—C17	125.26 (15)
C1—C2—C4	111.76 (12)	O1—C16—C17	111.84 (14)
C10—C2—C4	108.21 (11)	C18—C17—C22	119.12 (15)
C1—C2—C3	106.85 (13)	C18—C17—C16	122.13 (14)
C10—C2—C3	111.38 (13)	C22—C17—C16	118.74 (14)
C4—C2—C3	108.22 (13)	C19—C18—C17	119.90 (15)
C2—C3—H3A	109.5	C19—C18—H18	120.0
C2—C3—H3B	109.5	C17—C18—H18	120.0
H3A—C3—H3B	109.5	C20—C19—C18	120.24 (16)
C2—C3—H3C	109.5	C20—C19—H19	119.9
H3A—C3—H3C	109.5	C18—C19—H19	119.9
H3B—C3—H3C	109.5	C21—C20—C19	120.16 (16)
C9—C4—C5	116.42 (14)	C21—C20—H20	119.9
C9—C4—C2	120.38 (14)	C19—C20—H20	119.9
C5—C4—C2	123.19 (14)	C20—C21—C22	120.16 (16)
C4—C5—C6	121.80 (16)	C20—C21—H21	119.9
C4—C5—H5	119.1	C22—C21—H21	119.9
C6—C5—H5	119.1	C21—C22—C17	120.39 (16)
C7—C6—C5	119.62 (16)	C21—C22—H22	119.8
C7—C6—H6	120.2	C17—C22—H22	119.8
C5—C6—H6	120.2	O4—C23—O3	122.57 (14)
C6—C7—C8	120.34 (15)	O4—C23—C24	125.30 (15)
C6—C7—O1	116.81 (15)	O3—C23—C24	112.12 (14)
C8—C7—O1	122.56 (16)	C29—C24—C25	119.14 (15)
C7—C8—C9	119.25 (16)	C29—C24—C23	117.88 (15)
C7—C8—H8	120.4	C25—C24—C23	122.95 (15)
C9—C8—H8	120.4	C26—C25—C24	120.12 (16)
C8—C9—C4	122.53 (16)	C26—C25—H25	119.9
C8—C9—H9	118.7	C24—C25—H25	119.9
C4—C9—H9	118.7	C27—C26—C25	120.34 (19)
C15—C10—C11	116.33 (14)	C27—C26—H26	119.8
C15—C10—C2	123.36 (15)	C25—C26—H26	119.8
C11—C10—C2	120.28 (14)	C26—C27—C28	120.43 (17)
C12—C11—C10	122.32 (15)	C26—C27—H27	119.8
C12—C11—H11	118.8	C28—C27—H27	119.8
C10—C11—H11	118.8	C27—C28—C29	119.79 (17)
C13—C12—C11	119.27 (16)	C27—C28—H28	120.1
C13—C12—H12	120.4	C29—C28—H28	120.1
C11—C12—H12	120.4	C24—C29—C28	120.17 (17)
C12—C13—C14	120.73 (15)	C24—C29—H29	119.9
C12—C13—O3	118.80 (15)	C28—C29—H29	119.9

### Hydrogen-bond geometry (Å, °)

**Table:**

*D*—H···*A*	*D*—H	H···*A*	*D*···*A*	*D*—H···*A*
C26—H26···Cg2^i^	0.93	2.86	3.743 (2)	160

Symmetry codes: (i) −*x*+1, −*y*+1, −*z*+1.
